# Kinetics of T cell response in the testes and CNS during experimental autoimmune encephalomyelitis: Simultaneous blood-brain and -testis barrier permeability?

**DOI:** 10.22038/ijbms.2019.34510.8185

**Published:** 2019-07

**Authors:** Nafiseh Pakravan, Ameneh Ghaffarinia, Shahram Parvaneh, Cyrus Jalili, Farhad Riazi-Rad

**Affiliations:** 1 Division of Immunology, Medical School, Alborz University of Medical Sciences, Karaj, Iran; 2 Medical Biology Research Center, Kermanshah University of Medical Sciences, Kermanshah, Iran; 3 Department of Anatomy, Medical School, Kermanshah University of Medical Sciences, Kermanshah, Iran; 4 Department of Immunology, Pasture Institute of Iran, Tehran, Iran

**Keywords:** Barrier, CFA, CNS, EAE, Immune privilege, T cell, Testes

## Abstract

**Objective(s):**

Multiple sclerosis (MS) and its animal model, experimental autoimmune encephalomyelitis (EAE), are regarded as autoimmune diseases of the central nervous system (CNS). The CNS, testes, and eyes are immune privileged sites. It was initially presumed that ocular involvement in EAE and infertility in MS are neural-mediated. However, inflammatory molecules have been detected in the eyes of animals affected by EAE. It prompted us to investigate if the testes may also be targeted by immune response during EAE.

**Materials and Methods:**

kinetics of T cell response was investigated in the CNS and testes in EAE at different clinical scores. IFN-γ, IL-4, IL-17, and FoxP3 mRNA expressions were considered as representatives of Th1, Th2, Th17, and Treg, respectively.

**Results:**

In CNS, IL-17 and IFN-γ were initially up-regulated and attenuated at the late phase of the disease. IL-4 and FoxP3 were markedly down-regulated, but IL-4 was then up-regulated at the late phase of the disease. In the testes, IFN-γ and IL-17 were diminished but increased at the late phase of the disease. FoxP3 was gradually increased from the initial step to the peak of the disease. IL-17/ IFN-γ showed a similar pattern between the CNS and testes. However, FoxP3 and IL-4 expression appeared to have different timing patterns in the CNS and testes.

**Conclusion:**

Given the permeability in blood-retina/brain/CSF barrier by complete Freund’s adjuvant, the pattern of T cells may be changed in the testes during EAE as a consequence of the blood-testis barrier permeability. More research is required to explore the connection between immune privileged organs.

## Introduction

The concept of “immune privilege” has traditionally denoted tissues or sites where graft rejection is delayed or absent, such as the eye, the central nervous system (CNS), and the testes. It has become evident that immune privilege is a consequence of multiple mechanisms involving active regulation of antigen-speciﬁc immune responses within the privileged tissue. However, the immune system can attack immune privileged tissues in some circumstances, e.g., multiple sclerosis (MS), autoimmune orchitis, and autoimmune uveitis. Indeed, immune-privileged tissues are tissues in which localized immune responses are actively suppressed. Therefore, immune privilege does not mean that this tissue is absent from immune response, but rather that the regulatory mechanisms are active (1-3). These mechanisms have been shown to involve the localized blood barrier, altered major histocompatibility antigen expression, expression of the immunoregulatory non-classical MHC antigens, active immunosuppression mechanisms, and induction of antigen-speciﬁc immunosuppressive lymphocytes, especially CD4+ CD25+FoxP3+Treg cells (2, 3). 

In the normal state of the CNS, blood-brain barrier (BBB) and blood-cerebrospinal fluid (CSF) barrier (BCSFB) combined with the lack of an apparent lymphatic system, low constitutive levels of major histocompatibility antigen (MHC), molecules, local production of suppressive factors, limited numbers of professional antigen-presenting cells, and natural FoxP3^+^ regulatory T cells (Treg) cause the CNS environment to remain inherently hostile to activated adaptive immunity (2). Most specific immune-regulatory mechanisms to testicular antigens include the blood-testis barrier, altered expression of MHC, testicular microenvironment, and immunosuppressive elements such as Fas ligand and indoleamine 2,3 dioxygenase (3). Similarly, under physiological circumstances, blood-retina barrier (BRB), absence of lymphatic drainage of the interior of the intact globe, low constitutive levels of MHC molecules, an immunosuppressive ocular microenvironment, constitutive expression of apoptotic molecules on ocular cells help generate regulatory T cells (1). Previous studies demonstrated simultaneous encephalomyelitis and uveitis in experimental autoimmune encephalomyelitis (EAE), the experimental model for MS (4-6). On the other hand, the testes are affected in patients with MS and the onset of MS in men coincides with the beginning of the decline in bioavailable testosterone in approximately 50% of patients with MS. Sexual dysfunction can also develop in approximately 47–80% of patients with MS at the later stages of the disease (7-12). Also, spinal cord injury can cause infertility. Accordingly, we have observed regression of the testes and seminal vesicles in EAE (13-15). Considering the results demonstrating simultaneous encephalomyelitis and uveitis, we hypothesized that the T cell pattern could change in the brain and retina as well as the testes in EAE. It prompted us to investigate the trends of T cell immune response represented by IFN-γ, IL-17, IL-4, and FoxP3 in the CNS and testes during the course of EAE. 

## Materials and Methods

***Animal breeding***

Lewis rats were originally purchased from the* Darou Pakhsh*, Tehran, Iran. All animal, were given free access to food and water, maintained in light- and temperature-regulated rooms, and housed for one week before experiments. All experiments were done according to Animal Care and Use Protocol of Alborz University of Medical Sciences. 

***Experimental group and study design***

Six experimental groups were considered including one group as normal and five groups as animals affected by EAE at scores 1 to 5 (each n=5). EAE-induced animals were followed-up until they reached the considered score and then sacrificed. To do so, the animals were anesthetized with a ketamine and xylazine mixture and then perfused transcardially with cold PBS. Then, the CNS and testes of the animals were isolated, snap-frozen in liquid nitrogen, and stored at -80 ^°^C until use for total RNA extraction and measurement of cytokine levels. 

***EAE induction and clinical evaluation***

EAE was induced as described before (13-15). Briefly, rats between 8–9 weeks of age were immunized subcutaneously with 200 μl (100 μl on each of left and right sides of lower back) of a homogenate of equal volumes of a 50% suspension of guinea pig spinal cord and complete Freund’s adjuvant (CFA) (Difco, Germany) (1:1, v/v), containing 4 mg/ml *Mycobacterium tuberculosis* H3 RA (Difco Labs, Detroit, MI). Each rat received 50 μg guinea pig spinal cord and 400 μg *M. tuberculosis* H3 RA. 

The animals were daily weighed, and clinical signs of disease were evaluated. The different clinical signs after EAE induction were scored as follows: score 0, no symptoms; score 0.5, loss of tonicity of the distal portion at the tail or tail weakness; score 1, complete tail paralysis; score 2, mild paresis of hind limbs; score 3, complete paralysis of one hind limb; score 4, bilateral hind limb paralysis; score 5, complete paralysis (tetraplegia), urinary and/or fecal incontinence. 

***Real-time PCR***

Total RNA was extracted from each frozen brain, spinal cord, and testes using Trizol reagent (Invitrogen) according to a standard protocol. RNA concentrations were determined by a UV spectrophotometer (NanoDrop 2000c, Eppendorf) to make sure that the ratio at the absorbance of 260 nm to 280 nm wavelength (A260/A280) was 1.8–2.0. 

Expression of mRNA for β-actin, IFN-γ, IL-4, IL-17, and FoxP3 was determined using Rotor-Gene 6000™ (Corbett Research, Australia) thermocycler and SYBR®Premix Ex Taq™II Real-Time PCR Master Mix (Takara, Japan) in a final volume of 10 µl, according to the manufacturer’s instructions. Assay of the samples was in triplicate. The sequences of primers were forward 5’-aggccaaccgtgaaaagatg-3’ and reverse 5’-accagaggcatacagggacaa-3’ for β-actin and forward 5’- ccacggagaacgagctcatc-3’and reverse 5’- gagaaccccagacttgttcttca-3’ for IL-4, forward 5’-gggaagttggaccaccacat-3’ and reverse 5’-ttctccacccggaaagtgaa-3’ for IL-17, forward 5’-gaaagacaaccaggccatcag-3’ and reverse 5’-tcatgaatgcatccttttttg-3’ for IFN-γ, and forward 5’-cgggagagtttctcaagcac-3’ and reverse 5’- ggagctcttgtccactgagg-3’ for FoxP3. The efficiencies for primers used in the study varied between 95% and 105%. Primer pairs were validated to ensure the correct size of the PCR product and the absence of primer dimers. Thermocycler conditions included an initial step at 50 ^°^C for 5 min and a step at 95 ^°^C for 10 min. This was followed by a two-step PCR program at 95 ^°^C for 15 sec and 60 ^°^C for 60 sec for 40 cycles. The β-actin gene was chosen as an internal control against which mRNA expression of the target gene was normalized. The resultant gene expression level was presented as 2^-ΔCt^, in which ΔCt was the difference between Ct values of the target gene and β-actin. 

***Statistical analysis***

Data are shown as mean±SEM and statistically were analyzed using the GraphPad Prism statistical package and assessed by one-way ANOVA. In all cases, *P*-values less than 0.05 were considered statistically significant.

## Results

**Figure 1:**
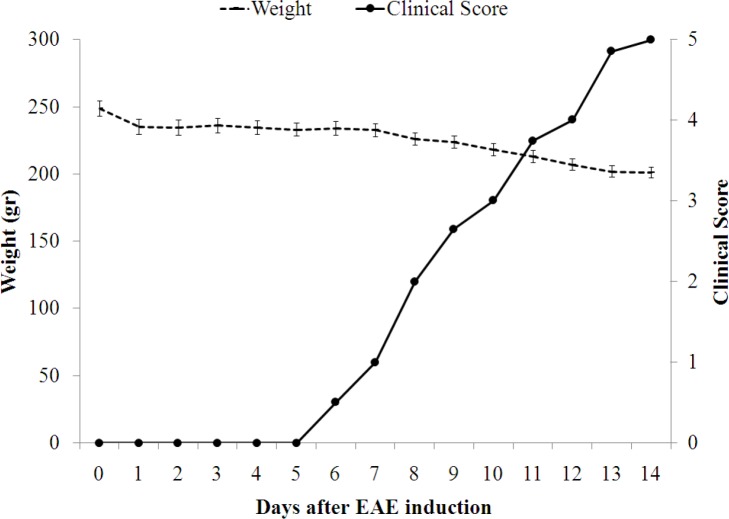
Timing of clinical scores and weight loss from day 1 to 14 after the immunization. To induce EAE, male Lewis rats were immunized subcutaneously with a suspension of guinea pig spinal cord and complete Freund's adjuvant (CFA). Clinical score and weight were measured daily from the day of disease induction. From the animals in which the disease was induced, five animals at each score were selected. Therefore, SEM is not applicable to the clinical score

In line with our previous report, the animals developed an acute monophasic disease with a reproducible and homogenous pattern (14). The disease which appeared with 100% incidence consisted of 5 stages, as explained in the previous section. EAE was induced in thirty animals who were monitored for 14 days to reach clinical scores 1 to 5. At each clinical score, five animals were sacrificed, and the CNS and testes were removed. As Figure 1 shows, initial signs of the disease (score 1) appeared on day 6, and afterward the disease flared up. On day 0 (before immunization), 7, 8, 10, 12, and 14 healthy animals and those at scores 1, 2, 3, 4, and 5 were chosen, sacrificed, and the CNS and testes were removed. The changes in expression of genes associated with EAE were estimated during the course of the disease from 0 or healthy state, to 5 or peak of the disease in the brain, spinal cord, and testes. Since EAE is a T cell-mediated disease, we investigated the changes in expression of IFN-γ, IL-4, IL-17, and FoxP3 as representative of Th1, Th2, Th17, and natural Tregs, respectively. 

**Figure 2:**
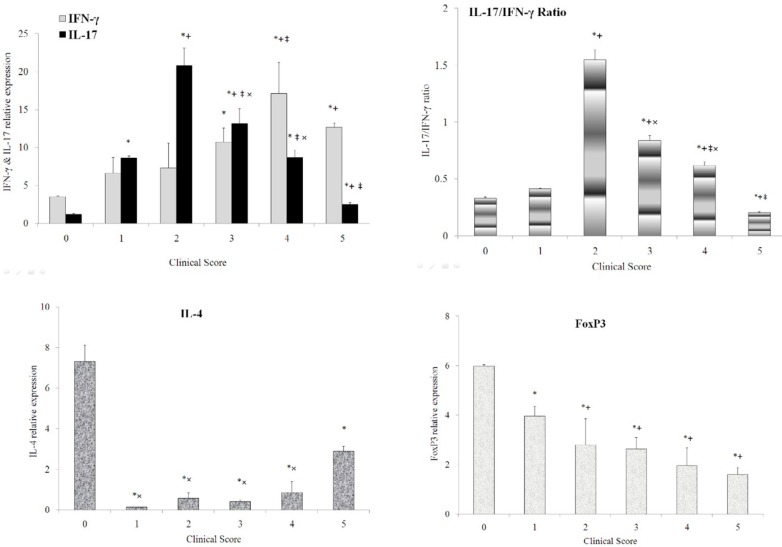
Kinetics of IFN-γ (a), IL-17 (a), IL-17/IFN-γ ratio (b), IL-4 (c), and FoxP3 (d) expression in the brain of normal rats and rats affected with EAE scoring from 1 to 5. IFN-γ (a) expression was significantly increased in animals at scores 3, 4, and 5 compared with the normal animals. IFN-γ expression in score 4 animals was also markedly up-regulated comparing to score 1 animals and showed a partially significant increase in score 4 and 5 animals compared to score 2 animals. IL-17 (a) level was markedly increased in score 1, 2, 3, 4, and 5 animals compared with normal animals. There was also significant down-regulation of IL-17 expression in score 5 animals compared with score 1, 2, 3, and 4 animals. In addition, there was a significant difference in IL-17 expression between the score 1 animals and the score 2 and 3 animals. IL-17 expression also showed a significant difference in score 2 animals with score 3 and 4 animals. IL-17/ IFN-γ ratio (b) significantly increased in the score 2 animals and showed a decreasing trend as the disease progressed. Level of IL-4 (c) expression was observed to be significantly attenuated in score 1, 2, 3, 4, and 5 animals compared with the normal animals. However, IL-4 expression in score 5 animals showed a partially significant increase compared to score 1, 2, 3, and 4 animals. However, there was no significant difference in IL-4 expression among score 2, 3, and 4 animals. FoxP3 (d) expression was observed to be significantly attenuated in score 1, 2, 3, 4, and 5 animals compared with the normal animals. It markedly dropped in score 2, 3, 4, and 5 animals compared with score 1 animals. However, there was no significant difference in FoxP3 expression between score 2, 3, and 4 animals. Data are presented as mean±SEM. (*) represents significant difference with the normal animals. (+) represents significant difference with score 1 animals. (‡) represents significant difference with the score 2 animal. (×) represents significant difference with score 5 animals

In the brain (Figure 2), IFN-γ expression (Figure 2a) was significantly increased in animals at scores 3, 4, or 5 compared with the normal animals. IFN-γ expression in score 4 animals was also markedly up-regulated comparing to score 1 animals and showed a partially significant increase (*P*=0.06) in score 4 and 5 animals compared to score 2 animals. Regarding IL-17 (Figure 2a), it showed an increasing trend and then decreased comparing to normal animals. IL-17/ IFN-γ ratio (Figure 2b) significantly increased in the brains of the score 2 animals and showed a decreasing trend as the disease progressed. Expression of IL-4 and FoxP3 was further explored as representative of Th2 response (16) and naturally occurring regulatory T cells, respectively. Level of IL-4 (Figure 2c) and FoxP3 expression (Figure 2d) was observed to be significantly attenuated in animals affected by the disease compared with the normal animals. However, IL-4 expression showed a partially significant increasing trend during the disease (0.05<*P*<0.1). In addition, FoxP3 expression markedly decreased in animals affected by the disease. 

**Figure 3.**
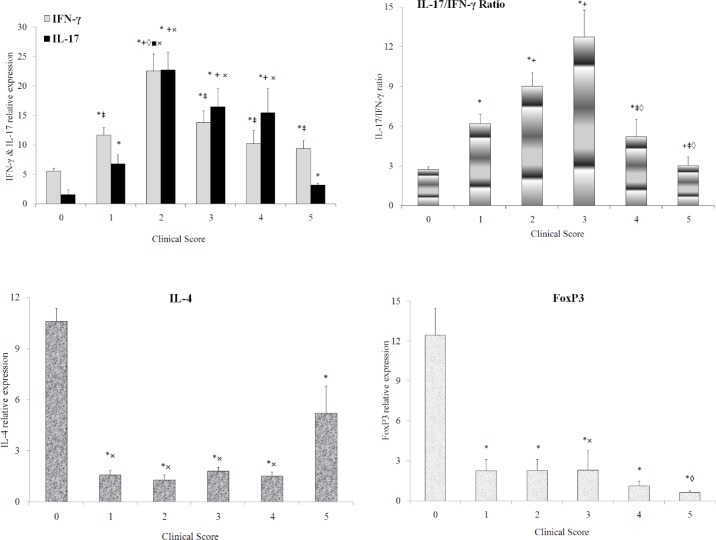
Trend of IFN-γ (a), IL-17 (a), IL-17/IFN-γ ratio (b), IL-4 (c), and FoxP3 (d) level in the spinal cord of healthy and EAE-induced rats scored from 1 to 5. IFN-γ (a) expression was significantly increased in score 1 to 5 animals comparing to the normal animals. IFN-γ expression in score 2 animals was also markedly up-regulated comparing to score 1, 3, 4, or 5 animals. IL-17 (a) level was markedly increased in score 1, 2, 3, and 4 animals compared with normal animals. The difference between normal animal and animals at score 5 was partially significant. There was also significant down-regulation of IL-17 expression in score 5 animals compared with score 2, 3, and 4 animals. Nevertheless, there was a significant difference in IL-17 expression between the animals at score 1 comparing to score 2, 3, and 4 animals. IL-17 expression was also markedly lower in score 1 animals than score 3 and 4 animals. IL-17/ IFN-γ ratio (b) significantly increased in the score 1 animals and peaked in score 3 animals and then showed a decreasing trend as the disease progressed. Level of IL-4 (c) expression was significantly attenuated in score 1, 2, 3, 4, and 5 animals compared with the normal animals. However, IL-4 expression in score 5 animals showed a marked increase compared to score 1, 2, 3, and 4 animals. FoxP3 (d) expression showed a significant down-regulation in score 1 to 5 animals compared with normal animals. However, there was a significant decline in FoxP3 expression in score 5 animals comparing to score 3 animals. Data are presented as mean±SEM. (*) represents significant difference with the normal animals. (+) represents significant difference with score on 1 animals. (‡) represents significant difference with the score 2 animal. (◊) represents significant difference with score 3 animals. (■) represents significant difference with score 4 animals. (×) represents significant difference with score 5 animals

In the spinal cord (Figure 3), IFN-γ expression (Figure 3a) showed a significant increasing trend, peaking at score 2, in the affected animals comparing to the normal animals. IL-17 expression (Figure 3a) showed a similar pattern as to IFN-γ expression. IL-17/ IFN-γ ratio (Figure 3b) had an increasing trend up to score 3 and then decreased as the disease progressed. Level of IL-4 expression (Figure 3c) was significantly decreased in animals affected by the disease. However, IL-4 expression showed a marked increase at the end of the disease (score 5). FoxP3 expression (Figure 3d) showed a significant decreasing trend during the disease. 

**Figure 4:**
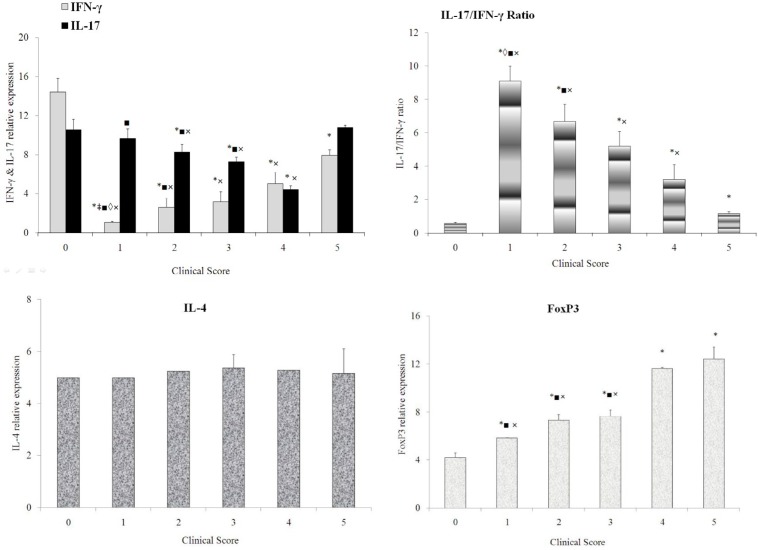
Trend of IFN-γ (a), IL-17 (a), IL-17/IFN-γ ratio (b), IL-4 (c), and FoxP3 (d) level in the testes of normal and EAE-induced rats scored from 1 to 5. IFN-γ (a) level was significantly attenuated in score 1 to 5 animals relative to normal animals. However, IFN-γ expression showed an increasing trend from the score 1 animals towards the score 5 animals. IL-17 (a) expression showed a decreasing trend from the score 1 animals towards the score 4 animals. However, it was increased in the score 5 animals. A significant difference was observed between score 3 and 4 animals compared with the normal animals. IL-17 expression in the score 1, 2, 3, and 5 animals was significantly higher than that of the score 4 animals. In addition, the score 2 and 3 animals expressed a significantly higher level of IL-17 than the score 5 animals. IL-17/ IFN-γ ratio (b) significantly increased at the beginning of the disease but showed a decreasing trend onward. IL-4 (c) showed no significant change. FoxP3 (d) expression was gradually increased as the disease progressed. Data are presented as mean±SEM. (*) represents significant difference with the normal animals. (+) represents significant difference with score 1 animals. (‡) represents significant difference with the scored 2 animal. (◊) represents significant difference with score 3 animals. (■) represents significant difference with score 4 animals. (×) represents significant difference with score 5 animals

In the testes (Figure 4), expression of IFN-γ (Figure 4a) was significantly decreased in the affected animals by the disease relative to normal animals. However, IFN-γ expression showed an increasing trend during the disease. IL-17 (Figure 4a) expression showed a gradually decreasing trend from the score 1 animals towards the score 4 animals and then increased at the end of the disease (score 5). IL-17/ IFN-γ ratio (Figure 4b) significantly increased in the score 1 animals and then showed a decreasing trend as the disease progressed. IL-4 level (Figure 4c) did not show a significant difference among normal and diseased animals. FoxP3 expression (Figure 4d) was significantly increased as the disease progressed comparing with normal animals. 

## Discussion

The testes and the CNS are both immune privileged sites with analogous mechanisms that operate in order to maintain immune privilege. These mechanisms have been shown to involve the localized blood barrier, altered major histocompatibility antigen expression, expression of the immunoregulatory non-classical MHC antigens, active immunosuppression mechanisms, and induction of antigen-speciﬁc immunosuppressive lymphocytes, especially CD4+ CD25+FoxP3+Treg cells (3, 17). It is well-established that systemic disorders can have inhibitory effects on male reproductive function (18). Therefore, this study aimed to examine the pattern of T cells in the testes and compare it with the pattern in the CNS during the course of EAE. Results on the kinetics of Th1 and Th17 cell pattern in the CNS are consistent with a previous result (19-21). The difference in the pattern of IFN-γ and IL-17 between the brain and the spinal cord may be an indication of unexplained preferential targeting of inflammation to the spinal cord in EAE (14, 22). The ratio of IL-17/ IFN-γ is also consistent with a previous report regarding the preferential homing pattern of Th17 and Th1 cells in the brain and spinal cord during development and later time points of EAE (23). The pattern of IL-4 expression and its ameliorating effect during recovery is also consistent with a previous report (16). Consistently, considering the critical role of IL-4 in memory and learning (24) along with the learning and memory dysfunction in MS and EAE (25, 26), in this study IL-4 was significantly decreased at the beginning and during the course of the disease but increased at the late phase of the disease. 

In contrast to the CNS, in the testes, IFN-γ expression markedly reduced at the onset of clinical signs and then gradually increased and peaked at the height of the disease (score 5). IL-17 expression showed a decreasing pattern during the course of the disease but returned to the normal level at the late time points of the disease. A previous report demonstrated that there is a kinetic of this cytokine in the CNS (19-21, 23) and this article shows that such a fluctuation is observed in both the CNS and testes. Notably, IL-17/IFN-γ ratio was significantly increased in both the CNS and testes, though with different timing patterns. The difference in the patterns of IFN-γ and IL-17 between the CNS and testes may be due to different physiologic roles of these cytokines depending on the environmental cells (27-30). However, the regulatory role of IFN-γ in both organs and its role as a master regulator of IL-17 level should also be taken into account (29, 30). Accordingly, IL-4, which has a physiologic role in the CNS (24), significantly decreases at the onset of clinical signs and remains low during the course of disease with a rise late in the disease. However, IL-4 level in the testes remained unchanged and appeared with no clear relation to the disease, which is consistent with other circumstances in the testes (31). In addition, the pattern of FoxP3 expression differed between the CNS and testes. While FoxP3 expression had a decreasing trend in the CNS, it appeared to have an increasing trend in the testes. The pattern of FoxP3 expression in the CNS was consistent and similar to the previous reports and in the testes was somehow similar to the experimental autoimmune orchitis (32, 33). Our results regarding CNS only show the validity and reliability of our results. The similarity in the T cell pattern in experimental autoimmune orchitis (33) and testes in EAE (our study) suggests that there seems to be some similarity between these conditions. In other words, although with different degree and pattern, presumably, there is an inflammatory response in the testes during EAE comparable with the situation in experimental autoimmune orchitis. However, the increasing trend of FoxP3 expression in the testes is also consistent with a previous study (35). This may be a local compensatory mechanism in order to fight the autoimmune processes or maybe because of Treg dysfunction, as seen in other circumstances (34, 35). The difference in FoxP3 expression pattern between the CNS and testes may be because of different magnitude of immune response and subsequently epitope spreading in the CNS (21). In addition, considering the role of testosterone in the expansion of the CD4+CD25+ Treg, it can be conceived that production of testosterone may increase as the disease recovers, leading to a Treg increase (3). Altogether, the anti-inflammatory mechanism including Treg and Th2 may be activated in the CNS and the testes in a different timing pattern. This may also be due to different favor of endothelial cells in the blood-brain or the blood-testis barriers on the migration of Treg or Th2 cells (36). 

Hypogonadism and reduced production of testosterone by Leydig cells have been reported in male MS patients and mice with EAE (11, 37, 38). Considering the immunosuppressive effect of testosterone, administration of testosterone supplements to MS patients has been suggested as a novel therapeutic approach (39, 40). Accordingly, treatment with agents such as D-aspartate, which ameliorates EAE, causes testosterone production in the testes and brain (41, 42). This is consistent with the concomitant decrease of the testosterone level and marked loss of myelinated nerve fibers (43). Interestingly, testicular-associated immune deviation is another sign of the immunosuppressive effect of normal testicular constituent in creating tolerance against autoimmunity (44, 45). Results of this study initially may raise the idea of possible antigenic similarity between the CNS itself with the testes. However, experiments based on the phenomenon mentioned above known as testicular-associated immune deviation make the possibility of such an assumption less likely. On the other hand, considering the critical role of CFA in experimental autoimmune disease induction, it is worth noting that different studies have reported that CFA causes increased permeability in the blood barrier with the brain, cerebrospinal fluid, retina, and testis (46-48). For example, administration of CFA in experimental autoimmune arthritis leads to decreased testosterone level (49-51), a condition similar to infertility mediated by testicular damage (52). Altogether, from the results mentioned above along with the results of this study, it is conceivable that CFA may vastly affect all of the barriers in the immune privileged sites (generalized permeability). Considering the presence of tight junctions in immune privileged sites, there are antigens in tight junctions shared by the CNS, eye, and testes (53, 54). This is consistent with previous results indicating infertility mediated by inflammation due to direct actions on the activity of the epithelial cells resulting in suppression of steroidogenesis (3, 46). The Leydig cells making testosterone are located next to tight junctions and Sertoli cells (3). This makes the testes an inconvenient microenvironment for normal production of testosterone. More research is required to unravel the immunologic aspect of Freund’s adjuvant and its effect on different tissues.

## Conclusion

This study demonstrated that the kinetics of T cell response concomitantly changed in the CNS and testes in EAE. Considering the effect of CFA on the blood barrier with the brain, cerebrospinal fluid, retina, and testis (46-48), the changes of T cell pattern in the testes during EAE may be a consequence of blood-testis barrier permeability. Therefore, the changes in fertility and testosterone level in MS/EAE may also be primarily because of blood-testis barrier permeability, similar to infertility mediated by testicular damage (52). In addition, the down-regulatory mechanism of inflammation including Treg and Th2 may be activated with different timing patterns in the inflamed CNS and testes. Considering the mass attack towards immune privileged sites (CNS, eye, and testes) in EAE, there may be some antigens in the blood-brain, - cerebrospinal fluid, -testis, and -retina barrier shared by these organs. This study for the first time concomitantly looks at and compares the kinetics of T cell response in the CNS & testes and suggests a situation similar to autoimmune orchitis for testes in EAE. More research is required to explore the possible interconnection between these organs.

## References

[1] Foster CS, Kothari S, Anesi SD, Vitale AT, Chu D, Metzinger JL (2016). The ocular immunology and uveitis foundation preferred practice patterns of uveitis management. Surv Ophthalmol.

[2] Hussain RZ, Hayardeny L, Cravens PC, Yarovinsky F, Eagar TN, Arellano B (2014). Immune surveillance of the central nervous system in multiple sclerosis- relevance for therapyand experimental models. J Neuroimmunol.

[3] Zhao S, Zhu W, Xue S, Han D (2014). Testicular defense systems: immune privilege and innate immunity. Cell Mol Immunol.

[4] Adamus G, Manczak M, Machnicki M (2001). Expression of CC chemokines and their receptors in the eye in autoimmune anterior uveitis associated with EAE. Invest Ophthalmol Vis Sci.

[5] Manczak M, Jiang S, Orzechowska B, Adamus G (2002). Crucial role of CCL3/MIP-1alpha in the recurrence of autoimmune anterior uveitis induced with myelin basic protein in Lewis rats. J Autoimmun.

[6] Segal BM, Raine CS, McFarlin DE, Voskuhl RR, McFarland HF (1994). Experimental allergic encephalomyelitis induced by the peptide encoded by exon 2 of the MBP gene, a peptide implicated in remyelination. J Neuroimmunol.

[7] Fode M, Krogh-Jespersen S, Brackett NL, Ohl DA, Lynne CM, Sønksen J (2012). Male sexual dysfunction and infertility associated with neurological disorders. Asian J Androl.

[8] Guo ZN, He SY, Zhang HL, Wu J, Yang Y (2012). Multiple sclerosis and sexual dysfunction. Asian J Androl.

[9] Noseworthy JH, Lucchinetti C, Rodriguez M, Weinshenker BG (2000). Multiple Sclerosis. N Engl J Med.

[10] Prévinaire JG, Lecourt G, Soler JM, Denys P (2014). Sexual disorders in men with multiple sclerosis: evaluation and management. Ann Phys Rehabil Med.

[11] Safarinejad MR (2008). Evaluation of endocrine profile, hypothalamic-pituitary-testes axis and semen quality in multiple sclerosis. J Neuroendocrinol.

[12] Bove R, Musallam A, Healy BC, Raghavan K, Glanz BI, Bakshi R (2014). Low testosterone is associated with disability in men with multiple sclerosis. Mult Scler.

[13] Ghaffarinia A, Jalili C, Parvaneh S, Mir-Aghaee S, Pakravan N (2015). Damage of urinary/respiratory system and survival rate is affected by gender in EAE model of Lewis rat. Acta SciVet.

[14] Ghaffarinia A, Parvaneh S, Jalili C, Riazi-Rad F, Yaslianifard S, Pakravan N (2016). Immunomodulatory effect of chymotrypsin in CNS is sex-independent: evidence of anti-inflammatory role for IL-17 in EAE. Iran J Allergy Asthma Immunol.

[15] Pakravan N, Ghaffarinia A, Parvaneh S, Yaslianifard S, Jalili C, Riazi-Rad F (2015). Neuroimmunomodulation by allogeneic seminal vesicle fluid in CNS is sex-independent. Neurol Res.

[16] Bitan M, Weiss L, Reibstein I, Zeira M, Fellig Y, Slavin S, Zcharia E (2010). Heparanase upregulates Th2 cytokines, ameliorating experimental autoimmune encephalitis. Mol Immunol.

[17] Russo MV, McGavern DB (2015). Immune surveillance of the CNS following infection and injury. Trends Immunol.

[18] Hedger MP (2011). Immunophysiology and pathology of inflammation in the testes and epididymis. J Androl.

[19] Issazadeh S, Mustafa M, Ljungdahl A, Höjeberg B, Dagerlind A, Elde R (1995). Interferon gamma, interleukin 4 and transforming growth factor beta in experimental autoimmune encephalomyelitis in Lewis rats: dynamics of cellular mRNA expression in the central nervous system and lymphoid cells. J Neurosci Res.

[20] McCombe PA, Nickson I, Pender MP (1998). Cytokine expression by inflammatory cells obtained from the spinal cords of Lewis rats with experimental autoimmune encephalomyelitis induced by inoculation with myelin basic protein and adjuvants. J Neuroimmunol.

[21] Momcilović M, Miljković Z, Popadić D, Miljković D, Mostarica-Stojković M (2008). Kinetics of IFN-gamma and IL-17 expression and production in active experimental autoimmune encephalomyelitis in Dark Agouti rats. Neurosci Lett.

[22] Ghaffarinia, Jalili C, Riazi-Rad F, Mostafaie A, Parvaneh S, Pakravan N (2014). Anti-inflammatory effect of chymotrypsin to autoimmune response against CNS is dose-dependent. Cell Immunol.

[23] Murphy AC, Lalor SJ, Lynch MA, Mills KH (2010). Infiltration of Th1 and Th17 cells and activation of microglia in the CNS during the course of experimental autoimmune encephalomyelitis. Brain Behav Immun.

[24] Gadani SP, Cronk JC, Norris GT, Kipnis J (2012). IL-4 in the brain: a cytokine to remember. J Immunol.

[25] Kurkowska-Jastrzębska I, Swiątkiewicz M, Zaremba M, Cudna A, Piechal A, Pyrzanowska J (2013). Neurodegeneration and inflammation in hippocampus in experimental autoimmune encephalomyelitis induced in rats by one--time administration of encephalitogenic T cells. Neurosci.

[26] Rao SM, Leo GJ, Bernardin L, Unverzagt F (1991). Cognitive dysfunction in multiple sclerosis Frequency patterns and prediction. Neurol.

[27] Ajami B, Bennett JL, Krieger C, McNagny KM, Rossi FM (2011). Infiltrating monocytes trigger EAE progression, but do not contribute to the resident microglia pool. Nat Neurosci.

[28] Balabanov R, Strand K, Goswami R, McMahon E, Begolka W, Miller SD (2007). Interferon-gamma-oligodendrocyte interactions in the regulation of experimental autoimmune encephalomyelitis. J Neurosci.

[29] Lees JR, Golumbek PT, Sim J, Dorsey D, Russell JH (2008). Reginal CNS responses to IFNγ determine lesion localization patterns during EAEpathogenesis. J Exp Med.

[30] Riccioli A, Starace D, D’Alessio A, Starace G, Padula F, De Cesaris P (2000). TNF-alpha and IFN-gamma regulate expression and function of the Fas system in the seminiferous epithelium. J Immunol.

[31] Klein B, Haggeney T, Fietz D, Indumathy S, Loveland KL, Hedger M (2016). Specific immune cell and cytokine characteristics of human testicular germ cell neoplasia. Hum Reprod.

[32] Fletcher JM, Lalor SJ, Sweeney CM, Tubridy N, Mills KH (2010). T cells in multiple sclerosis and experimental autoimmune encephalomyelitis. Clin Exp Immunol.

[33] Jacobo P, Guazzone VA, Theas MS, Lustig L (2011). Testicular autoimmunity. Autoimmun Rev.

[34] Baráth S, Sipka S, Aleksza M, Szegedi A, Szodoray P, Végh J (2006). Regulatory T cells in peripheral blood of patients with mixed connective tissue disease. Scand J Rheumatol.

[35] Singh AM, Dahlberg P, Burmeister K, Evans MD, Gangnon R, Roberg KA (2013). Inhaled corticosteroid use is associated with increased circulating T regulatory cells in children with asthma. Clin Mol Allergy.

[36] Biernacki K, Prat A, Blain M, Antel JP (2001). Regulation of Th1 and Th2 lymphocyte migration by human adult brain endothelial cells. J Neuropathol Exp Neurol.

[37] Foster SC, Daniels C, Bourdette DN, Bebo BF Jr (2003). Dysregulation of the hypothalamic- pituitary-gonadal axis in experimental autoimmune encephalomyelitis and multiple sclerosis. J Neuroimmunol.

[38] Dane S, Timur H (2005). Sex-related differences in tuberculin reaction, free and total testosterone concentrations in patients with autoimmune disorders and controls. Int J Neurosci.

[39] Macció DR, Calfa G, Roth GA (2005). Oral testosterone in male rats and the development of experimental autoimmune encephalomyelitis. Neuroimmunomodul.

[40] Palaszynski KM, Loo KK, Ashouri JF, Liu HB, Voskuhl RR (2004). Androgens are protective in experimental autoimmune encephalomyelitis: implications for multiple sclerosis. J Neuroimmunol.

[41] Mellon SH, Griffin LD, Compagnone NA (2001). Biosynthesis and action of neurosteroids. Brain Res Rev.

[42] Nagata Y, Homma H, Lee JA, Imai K (1999). D-aspartate stimulation of testosterone synthesis in rat leydig cells. FEBS Lett.

[43] Marner L, Nyengaard JR, Tang Y, Pakkenberg B (2003). Marked loss of myelinated nerve fibers in the human brain with age. J Comp Neurol.

[44] Ditzian-Kadanoff R (1999). Testicular-associated immune deviation and prevention of adjuvant-induced arthritis by three tolerization methods. Scand J Immunol.

[45] Veräjänkorva E, Setälä N, Teros T, Salmi AA, Pöllänen P (2002). Testicular-associated immune deviation: flushing of the testicular lymph sinusoids induces immunosuppression and inhibits formation of EAE in SJL mice. Scand J Immunol.

[46] Brooks TA, Hawkins BT, Huber JD, Egleton RD, Davis TP (2005). Chronic inflammatory pain leads to increased blood-brain barrier permeability and tight junction protein alterations. Am J Physiol Heart Circ Physiol.

[47] Rabchevsky AG, Degos JD, Dreyfus PA (1999). Peripheral injections of Freund’s adjuvant in mice provoke leakage of serum proteins through the blood-brain barrier without inducing reactive gliosis. Brain Res.

[48] Reiber H, Suckling AJ, Rumsby MG (1963). The effect of Freund’s adjuvants on blood-cerebrospinal fluid barrier permeability. J Neurolog Sci.

[49] Carageorgiou HK, Stratakis CA, Damoulis PD, Varonost DD, Messari ID, Sideris ACh (2005). Reversible plasma testosterone levels reduction after gentamicin administration and freund’s adjuvant arthritis in rats. Indian J Physiol Pharmacol.

[50] Flake NM, Hermanstyne TO, Gold MS (2006). Testosterone and estrogen have opposing actions on inflammation-induced plasma extravasation in the rat temporomandibular joint. Am J Physiol Regul Integr Comp Physiol.

[51] Staykova M (2012). Rat model for poly-autoimmunity. Am J Immunol.

[52] Kukadia AN, Ercole CJ, Gleich P, Hensleigh H, Pryor JL (1996). Testicular trauma: potential impact on reproductive function. J Urol.

[53] Gow A, Southwood CM, Li JS, Pariali M, Riordan GP, Brodie SE, Danias J (1999). CNS myelin and Sertoli cell tight junction strands are absent in Osp/claudin-11 null mice. Cell.

[54] Morita K, Sasaki H, Fujimoto K, Furuse M, Tsukita S (1999). Claudin-11/OSP-based tight junctions of myelin sheaths in brain and Sertoli cells in testes. J Cell Biol.

